# Systemic and Mucosal Humoral Immune Response Induced by Three Doses of the BNT162b2 SARS-CoV-2 mRNA Vaccines

**DOI:** 10.3390/vaccines10101649

**Published:** 2022-10-01

**Authors:** Roberta Mancuso, Simone Agostini, Lorenzo Agostino Citterio, Debora Chiarini, Maria Antonia Santangelo, Mario Clerici

**Affiliations:** 1 IRCCS Fondazione Don Carlo Gnocchi ONLUS, 20148 Milan, Italy; 2 Department of Pathophysiology and Transplantation, University of Milan, 20122 Milan, Italy

**Keywords:** SARS-CoV-2, antibodies, mRNA vaccines, rehabilitation, humoral response, BNT162b/Pfizer/Comirnaty, mucosal response

## Abstract

BNT162b2 (BioNTech/Pfizer) was the first SARS-CoV-2 mRNA vaccine approved by the European Medicines Agency. We monitored the long-term humoral responses of healthcare workers (HCWs) who received three vaccine doses. A total of 59 healthcare workers were studied: 47 were never SARS-CoV-2-infected (naïve-HCWs), and 12 (infected-HCWs) recovered from COVID-19 before the first vaccine. Serum and saliva were collected at baseline (before the first dose), just before the second dose, 1, 3, 6, and 9 months after the second dose, and 10 days after the third vaccine. SARS-CoV-2-specific IgG and IgA were evaluated in serum and saliva, respectively, and the presence of neutralizing antibodies (NAb) was analyzed in serum. SARS-CoV-2-specific IgG peaked one month after the second vaccine in naïve-HCWs but right before this timepoint in infected-HCWs. IgG titers significantly decreased during follow-up and at month 9 were still detectable in 50% of naïve-HCWs and 90% of infected-HCWs. NAb were significantly decreased 6 months after the second vaccine in naïve-HCWs and 9 months after this dose in infected-HCWs. Salivary SARS-CoV-2-specific IgA titers were significantly higher in infected-HCWs and were undetectable 9 months after the second vaccine in 43% of the naïve-HCWs alone. The third vaccine greatly increased humoral IgG and mucosal IgA in both groups. Two BNT162b2 doses induced strong systemic and humoral immune responses; to note, these responses weakened over time, although they are more prolonged in individuals who had recovered from COVID-19. The third vaccine dose quickly boosts systemic and mucosal humoral responses.

## 1. Introduction

Severe acute respiratory syndrome coronavirus 2 (SARS-CoV-2) is a new single-stranded RNA virus responsible for the coronavirus disease 2019 (COVID-19) [1], a severe and potentially fatal disease responsible for the ongoing worldwide pandemic. In one of the most remarkable successes of scientific research, several vaccines against this virus became available within less than a year of the beginning of the pandemic. The nucleoside-modified mRNA vaccine BNT162b2 (Comirnaty), in particular, was developed by BioNTech/Pfizer and has been proven to be one of the most important tools to prevent the severe form of COVID-19. This vaccine is a lipid nanoparticle–formulated, nucleoside-modified RNA encoding the SARS-CoV-2 full-length spike glycoprotein. BNT162b2 administration showed 95% effectiveness in the general population against the severe disease and a favorable safety profile [2], supporting the emergency use authorization of this vaccine both in the USA [3] and Europe [4] for ≥16 years old subjects and more recently for adolescent [5] and children as well [6].

The humoral immune response to BNT162b2 in healthy uninfected people has been the main focus of previous studies [7,8,9,10]. Results showed that two doses of vaccine are needed to reach an effective and strong IgG antibody with limited involvement of IgM and IgA in serum [11]. Fewer data are available on the kinetics and persistence of vaccine-stimulated antibody responses, given that the vaccination campaign started at the end of 2020. In Italy and other European countries, high-risk healthcare professionals were prioritized to receive the first vaccine doses in December 2020. The organization of the vaccinal campaign in this country was extremely efficient, and by July 2022, more than 90% of the population over twelve years had received at least two doses of the vaccine [12].

SARS-CoV-2-specific humoral responses in infected, unvaccinated individuals have been associated with COVID-19 severity [13] and were shown to decline within 12 months after the onset of the disease [14]. Antibody titers declined more rapidly in subjects with mild/asymptomatic SARS-CoV-2 infection [15,16,17,18,19]. A decreased trend of humoral immunity is also evident in vaccinated individuals after the second vaccine dose [7,20]; however, scarce data exist analyzing humoral responses after the administration of a third dose of the vaccine [21,22], and even fewer data focus on the ability of this vaccine to generate mucosal humoral immune responses. 

We designed a prospective, longitudinal observational study in a cohort of healthcare workers (HCWs) who received three doses of the BNT162b2 vaccine; systemic and mucosal immune responses were analyzed before the first dose of the vaccine, as well as after the second and third dose of the vaccine to verify the magnitude, characteristics, and persistence of BNT162b2 vaccine-induced humoral immune responses.

## 2. Materials and Methods

### 2.1. Individuals and Sample Collection 

Fifty-nine healthcare workers of the Fondazione Don Gnocchi in Milan, Italy, were enrolled in the study. All these individuals received three doses of the Pfizer-BioNTech Comirnaty mRNA vaccine (BNT162b2), with the standard 3-week interval between the first and the second dose used in Italy.

**Figure 1:**
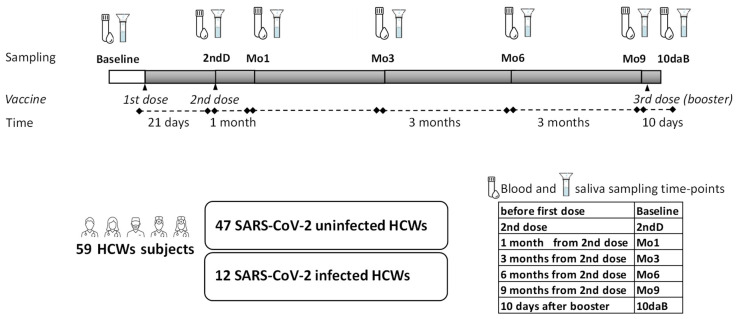
Overview of the study workflow. The schedule for BNT162b2 vaccine administration, the timing of blood and saliva sampling, and a scheme of groups of healthcare workers (HCWs) enrolled for this study.

Twelve of these subjects had recovered from mild COVID-19 disease before receiving the first dose of the vaccine (infected-HCWs). The other 47 (naïve-HCWs) did not show previous COVID-19. Twenty-three (40%) of the enrolled individuals were directly taking care of COVID-19 patients in the rehabilitative unit of Fondazione Don Carlo Gnocchi ONLUS, Milan, Italy. The other 36 individuals were researchers, technicians, and office workers. At the time of the study, none of the infected HCWs had residual symptoms. The study was carried out between 18 January 2021 and 27 December 2021. Whole blood and saliva were collected from all the enrolled subjects just before the administration of the first (baseline) and the second dose of vaccine (2nd D), 1 (Mo1), 3 (Mo3), 6 (Mo6), and 9 months (Mo9) after the second vaccine dose, and 10 days after the third vaccine dose (10daB) (schematized in Figure 1). Whole blood collected into sterile collection tubes with a gel separator (BD Vacutainer® SST™ II Advance tubes, Becton-Dickinson Biosciences, San Josè, CA, US) was allowed to clot at room temperature for 1 h and then centrifuged (2000*g* × 10’) to obtain serum. Aliquots of serum samples were stored at −80 °C for the analyses. Whole saliva samples collected by passive drool were immediately stored in aliquots at −80 °C until use. SARS-CoV-2-specific IgG, IgA, and neutralizing antibodies (NAb) were measured in serum, and virus-specific IgA was measured also in saliva, from all individuals. Nasopharyngeal swab samples collected monthly from all the subjects were routinely tested using a COVID-19 Antigen Rapid Test for the hospital surveillance program. The study conformed to the ethical principles of the Declaration of Helsinki; all subjects gave informed and written consent according to a protocol approved by the ethics committee of IRCCS Fondazione Don Carlo Gnocchi ONLUS (n#6_17/02/2021).

### 2.2. Immunological Analyses

SARS-CoV-2-specific IgG was measured in serum using a commercial chemiluminescence immunoassay (Access SARS-CoV-2 IgG), performed with an automated analyzer (Access Immunoassay Systems; Beckman Coulter, Brea, CA, USA), according to manufacturer’s instructions. In this assay, 20 µL of serum was added to reaction vessels with buffer and paramagnetic particles coated with recombinant receptor binding domain (RBD) of the S1 protein (Wuhan-Hu-1, wild type); IgG antibody titers were expressed as AU/mL (arbitrary units/mL; cut-off: 10 AU/mL). 

SARS-CoV-2-specific IgA in saliva or serum was measured using a commercial kit assay (RayBio, CliniSciences, Guidonia Monticello, Rome, Italy), according to standard protocol. Also in this case, wells are coated with recombinant RBD of the S1 protein (Wuhan-Hu-1, wild type). Saliva samples were diluted at 1:2, whereas serum was diluted at 1:500. Optical densities (OD) for each well were read on a plate reader (Sunrise, Tecan, Mannedorf, Switzerland) at 450 nm. IgA results were expressed as U/mL (units/mL; cut-off: 21.4 U/mL).

The presence of SARS-CoV-2-specific neutralizing antibodies (NAb) was assessed using a commercial SARS-CoV-2 Surrogate Virus Neutralization Test Kit (GenScript, CliniSciences) according to standard protocol. This kit mimics the virus-host interaction in an ELISA test using two key components: the horseradish peroxidase (HRP) conjugated recombinant viral spike (S) protein receptor-binding domain RBD fragment (HRP-RBD, Wuhan-Hu-1, wild type) and the human ACE2 receptor protein (hACE2). The protein-protein interaction between HRP-RBD and hACE2 can be blocked by neutralizing antibodies against the SARS-CoV-2 RBD. Sera were diluted 1:10. For each sample, optical densities (OD) at 450 nm were read on an absorbance plate reader (Sunrise, Tecan, Mannedorf, Switzerland). According to the manufacturer’s instruction, the percentage of inhibition was calculated by the formula: (1–ODsample/ODnegative control) × 100%; values <30% were considered as negative, i.e., absence or level of SARS-CoV-2 neutralizing antibody below the limit of detection of the assay. Previous validation studies demonstrate the high specificity/sensitivity of this assay and indicate that results from surrogate virus neutralization assays correlate highly with results from conventional live virus neutralization methods [23].

### 2.3. Virological Analyses

Viral RNA was extracted from saliva by a commercial kit (QIAmp Viral RNA, Qiagen GmbH, Hilden, Germany), following the manufacturer’s protocol.

SARS-CoV-2 viral RNA detection was performed by reverse transcription-droplet digital PCR (RT-ddPCR, QX200, Bio-Rad, Hercules, CA, USA), using the 2019-nCoV CDC ddPCR Triplex Probe assay (Bio-Rad SARS-CoV-2 ddPCR Test) that allow to detection in a single tube of two viral targets (in nucleocapsid region, N1 and N2) and one genomic control (human RNaseP gene). Synthetic RNA transcripts (Exact Diagnostic SARS-CoV-2 Standard) containing five gene targets (E, N, ORF1ab, RdRP, and S Genes of SARS-CoV-2) and human genomic DNA were processed as samples and used to assess the extraction procedure and as standard to quantify viral load. For each experiment, a negative control (no template) was included. Five μL of viral RNA were added to the master mix, which was emulsified with droplet generator oil (Bio-Rad) using a QX200 droplet generator. The droplets were loaded into a 96-well reaction plate and heat-sealed with an aluminum foil sheet (PX1, PCR plate sealer, Bio-Rad). Reverse transcription and amplification were then performed using T100 thermal cycler (Bio-Rad) with the following cycling parameters: 3 min at 25 °C, 60 min at 50 °C, 10 min at 95 °C, 40 cycles at 95 °C for 30-s, and 55 °C for 60 s, followed by 10 min at 98 °C and a hold at 4 °C. After PCR amplification, the plate was transferred to a QX200 droplet reader (Bio-Rad). Each well was queried for fluorescence to determine the number of positive events (droplets), and the results were displayed as dot plots and analyzed by QuantaSoft Analysis Pro Software (version 1.0.596). The RNA concentration was expressed as copies/μL. The detection limit is 0.625 copies/μL of reaction for both N1 and N2 genes. 

### 2.4. Statistical Analysis

Normally distributed continuous data were expressed as mean ± standard deviation, and comparisons among groups were analyzed by ANOVA test and Student t-test, when appropriate. Median and interquartile range (IQR: 25th and 75th percentile) was used for skewed continuous variables, and comparisons were analyzed by Kruskal-Wallis and Mann-Whitney U test, as appropriate, and with Wilcoxon signed-rank test for paired data. Correlations were assessed by Spearman’s rank tests. *p*-values corresponding to ≤0.05 were statistically significant. Regarding IgG analysis, we assigned an arbitrary value of 500 AU/mL for those that resulted in the upper limit of detection (>434 AU/mL). The statistical analyses were accomplished using commercial software (MedCalc Statistical Software version 14.10.2, Ostend, Belgium).

## 3. Results

### 3.1. Clinical Parameters

The study enrolled 59 healthcare workers (Table 1). Forty-seven of these individuals (15 men/32 women) were SARS-CoV-2 uninfected subjects (naïve-HCWs) as assessed by a negative serological test (IgG); the remaining 12 subjects (4 men/8 women) had a documented history of previous natural SARS-CoV-2 infection (infected-HCWs) (positive molecular nasopharyngeal swab and/or IgG serological tests for to SARS-CoV-2) occurring before the vaccination (median interval of 108 days; IQR: 80-218 days). One subject in this second group suffered from severe infection, while all the others were affected with mild or pauci-symptomatic infection. The two groups were comparable in age and gender. All the individuals were immunocompetent and in good condition before vaccination, and no one was taking immunosuppressive treatment. Comorbidity conditions were: hypertension (22%), autoimmune disease (22%), diabetes (17%), cancer (13%) and allergy (13%).

Vaccination was well tolerated in all enrolled subjects (no side effects or modest side effects: fever, headache, and fatigue for 1–2 days). All the analyzed saliva samples were negative for SARS-CoV-2 RNA molecular detection. During the study, no subject developed COVID-19.

### 3.2. Serum SARS-CoV-2 IgG and IgA Antibody Response after Vaccination

**Figure 2:**
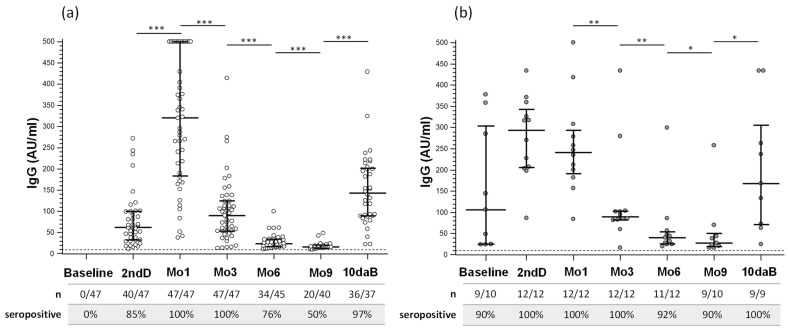
Serum IgG antibody response to SARS-CoV-2 vaccine. Serum IgG response to COVID-19 mRNA vaccine (Pfizer) measured in (**a**) SARS-CoV-2 uninfected healthcare workers (naïve-HCWs) and (**b**) SARS-CoV-2 previously infected-HCWs before the vaccination, at the time of second dose (2ndD), after one (Mo1), three (Mo3), six (Mo6) and nine months (Mo9), when the booster dose was administered, and ten days after booster (10daB). AU: arbitrary Units. * *p* ≤ 0.05; ** *p* ≤ 0.001; *** *p* ≤ 0.0001.

SARS-CoV-2-specific IgG concentrations are reported in Figure 2 and Supporting Information Table S1. At baseline, all the naïve-HCWs subjects were IgG seronegative, while 90% of infected-HCWs were seropositive. At the moment of the second dose of vaccine (2ndD), most of the uninfected subjects (40/47; 85%) and the entire group (12/12; 100%) of previously infected subjects resulted seropositive for SARS-CoV-2-specific IgG, and median IgG concentration was significantly increased in infected subjects compared to uninfected subjects (*p* < 0.0001) (see Supporting Information Figure S1).

One month after the second dose of vaccine (Mo1), the percentage of infected-HCWs (100%; *p* = 0.01 vs. 2ndD) and IgG titers significantly increased in naïve-HCWs (*p* < 0.0001 vs. 2ndD). At 3, 6, and 9 months after the second vaccine dose, IgG serum titers progressively declined (*p* < 0.0001 for all timepoints vs. 2ndD), and 50% of naïve-HCWs were seronegative 9 months after the 2nd vaccine dose.

A similarly decreasing trend in IgG titers was observed after the 2nd vaccine dose in infected-HCWs (Mo3, Mo6 vs. 2ndD: *p* < 0.001; Mo9 vs. 2ndD: *p* = 0.0039). Notably, though: (1) SARS-CoV-2-specific IgG serum concentrations 6 and 9 months after the second vaccine dose were significantly higher than that observed in naïve-HCWs (*p* = 0.014 and *p* = 0.0054 respectively) (see Supporting Information Figure S1), and (2) 90% of infected-HCWs were still seropositive 9 months after the second vaccine dose (*p* = 0.031 vs. HCWs).

The third vaccine dose significantly increased serum titers of SARS-CoV-2-specific IgG in all but one individual (the exception being a naïve-HCW) (*p* < 0.0001 in naïve-HCWs; *p* = 0.03 in infected-HCWs vs. Mo9); to note, ten days after the third vaccine dose (10daB), IgG titers in naïve-HCWs were significantly lower than those observed 1 month after the second vaccine dose (*p* < 0.0001). Whereas no gender-related differences were observed, IgG titers at the 2ndD, Mo1, and Mo3 time points were inversely correlated with age in naïve-HCWs.

IgA concentrations were measured in serum at baseline, 2nd dose, at Mo1, Mo9, and 10daB. The majority of subjects developed IgA after two doses of vaccine (89% naïve-HCWs and 100% of infected-HCWs); at 9Mo, almost all the infected-HCWs remained IgA positive, whereas IgA response was detected in only 35% of naïve-HCWs (*p* = 0.0031). Importantly the majority of subjects in both groups (86% naïve-HCWs and 91% of infected-HCWs) developed IgA response again after a booster dose. IgA concentrations were significantly higher in infected-HCWs compared to naïve-HCWs (*p* < 0.05; see Supporting Information Figure S1) at every time point, except for the time point following the booster dose (*p* = 0.08) (see Supporting Information Table S2). However, IgA in serum significantly increased in naïve-HCWs alone after 2nd dose (1Mo) and after the booster dose (10daB). A positive correlation between IgG and IgA concentration was found only at 10daB (*p* = 0.0003).

### 3.3. Neutralizing Antibodies Induced by Vaccination 

At baseline, SARS-CoV-2-specific neutralizing antibodies were detected in 70% of infected HCWs but in none of the naïve-HCWs. NAb could be detected in more than 70% of naïve-HCWs and infected-HCWs at the 2ndD time point. 100% of naïve-HCWs showed NAb in serum after one (Mo1) and three (Mo3) months from the second dose, declining to 87% at Mo9 after the second vaccine dose. All the infected naïve-HCWs were consistently NAb positives throughout the entire follow-up (Mo3, Mo6, Mo9).

Inhibitory activity (INH, expressed as % of inhibition of binding of RBD and ACE2) at the 2ndD was significantly higher in infected-HCWs (INH: 97.71) than in naïve-HCWs (INH: 56.02; *p* < 0.0001); it remained consistently elevated (INH over 80) until Mo6, when a more evident decline was observed in naïve-HCWs (INH: 65.47) than in infected-HCWs (INH: 74.38; *p* < 0.0001). All the subjects were positive for NAb after the third vaccine dose, which greatly boosted neutralizing activity both in naïve-HCWs (INH 83.7; vs. Mo9 months: *p* < 0.0001) and in infected-HCWs (96.9%) (Supporting Information Table S3).

Significant negative correlations were observed in naïve-HCWs between neutralizing activity (INH) and age at every time point (*p* < 0.05 in all cases) except for the 2ndD time point. SARS-CoV-2-specific INH and IgG titers in naïve-HCWs were significantly correlated at every time point (*p* < 0.0001 in all cases) except for Mo9 and 10daB. Notably, no associations were found between IgG, INH, and age in infected-HCWs. Serum IgA concentration and INH did not associate in both groups at every time point.

### 3.4. Salivary SARS-CoV-2 IgA Isotype Antibody Response after Vaccination

**Figure 3:**
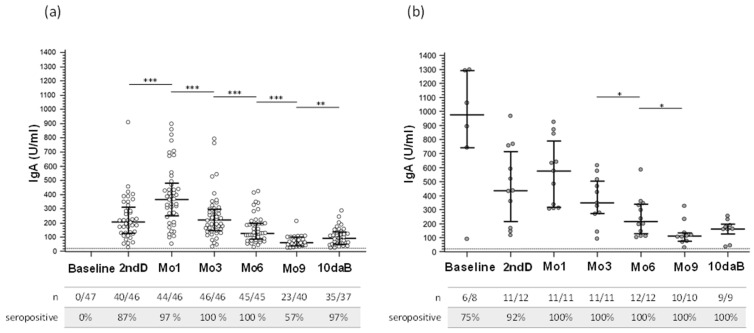
Salivary IgA antibody response to SARS-CoV-2 vaccine. Salivary IgA response to COVID-19 mRNA vaccine (Pfizer) measured in SARS-CoV-2 uninfected healthcare workers (*naïve-HCWs*) (**a**) and SARS-CoV-2 previously *infected-HCWs* (**b**) before the vaccination, at the time of second dose (2ndD), after one (Mo1), three (Mo3), six (Mo6) and nine months (Mo9), when the booster dose was administered, and ten days after booster (10daB). U: Units. * *p* ≤ 0.005; ** *p* ≤ 0.001; *** *p* ≤ 0.0001.

At baseline, SARS-CoV-2-specific salivary IgA antibodies were detectable in 6 infected-HCWs. At the 2ndD time point, salivary IgA could be observed in most naïve-HCWs (87%) and infected-HCWs (92%) (Supporting Information Table S4), indicating the ability of the Pfizer-BioNTech Comirnaty mRNA vaccine to elicit mucosal immunity. These antibodies remained detectable in all subjects of both groups until the Mo6 time point and could still be observed at Mo9 in 57% of naïve-HCWs and all infected-HCWs (100%; *p* = 0.01). The third vaccine dose significantly increased salivary IgA titers both in naïve-HCWs (*p* = 0.001). IgA titers were always significantly higher in infected-HCWs compared to naïve-HCWs (*p* < 0.05 at any time point vs. naïve-HCWs) (Figure 3 and Supporting Information Figure S1). Salivary IgA concentrations were significantly lower at every time point than those observed in serum samples (*p* < 0.0001). No correlations were observed between IgA detected in serum and saliva.

## 4. Discussion

This study monitors the humoral responses in a cohort of BNT162b2 fully vaccinated Italian healthcare workers by analyzing serum titers of SARS-CoV-2-specific IgG, IgA and neutralizing activity as a measure of antibodies effector function; mucosal immunity was evaluated as well by measuring salivary virus-specific IgA. The period of observation began just before the administration of the first dose of the vaccine. It was extended over a prolonged time after the second dose (more than 9 months), as well as one week after the third dose of vaccine, indicated by the Italian Ministry after at least four months from the completion of the primary. At baseline and during follow-up, viral RNA was undetectable in all individuals by ddPCR in saliva samples. The subjects enrolled in the study were routinely tested monthly for the presence of nucleocapsid protein by rapid antigen tests, and all tested negative. Notably, none of the HCWs developed COVID-19 after vaccination during the study. 

The first dose of the vaccine was highly effective in inducing IgG response, as most enrolled subjects became IgG seropositive before the second vaccine administration, scheduled 21 days after the first dose. IgG titers were significantly increased by the second dose of the vaccine and then decreased progressively over time. Thus, the majority of individuals still showed the presence of IgG titers 6 months after the 2nd vaccine dose, but the percentage of seropositive individuals well as median IgG titers were drastically reduced 9 months after the second vaccine dose. One of the findings of this study is that several individuals seroreverted within 9 months after the second dose of vaccine. The waning of the humoral response against SARS-CoV-2 up to 9 months after the second dose of the vaccine has been observed by several authors [8,24]. Many factors may contribute to this phenomenon, including the observations that: (1) this is an mRNA and not a protein-based or an attenuated-virus vaccine; (2) the spike antigen accumulates mutations over time; (3) the role played by individual characteristics, including age, sex, comorbidity, and genetic background, in determining the strength and the duration of vaccine-induced immune responses is still unclear. The potential role of each of these factors will need to be clarified. Finally, it cannot be excluded that at least some of the apparent seroreversion could be false negatives.

The third dose of vaccine was highly effective in re-boosting IgG titers. However, the median IgG concentration is lower than that observed 1 month after the second dose for both naïve-HCWs (10daB: median, 143 UA/mL; vs. 1Mo: 319: *p* < 0.0001) and infected-HCWs (10daB: 168 UA/mL; vs. 1Mo: 240 UA/mL, ns). Probably the limited period (10 days) after the vaccine booster did not allow IgG production to reach its peak; other experiments with longer follow-ups after the booster will be needed to clarify this puzzling observation.

A similar trend was observed when serum IgA was analyzed, even if a lower number (45%) of naïve-HCWs developed IgA after the first dose. Notably, previous natural infection strongly elicits the generation of serum IgA in infected-HCWs, as shown by the high concentration measured at baseline. Moreover, although the IgA titers were significantly higher in infected-HCWs compared to naïve subjects, such titers did not show significant differences after the two vaccine doses in infected-HCWs, in accordance with previous papers [25,26]. Finally, the booster dose was very effective in up-regulating IgA production, suggesting a different kinetic response to the vaccine in IgA and IgG; this is in line with what had been previously observed [27].

This is extremely important, as humoral response induced by the third dose of the BNT162b2 vaccine was shown to be a major contributor to immune protection against COVID-19-associated severe outcomes and mortality [21,28,29]. Not surprisingly, the effect of the different vaccine doses was more potent in those individuals who had been previously SARS-Cov-2 infected.

These last data confirm that the response to the first dose of vaccine is more rapid and robust in subjects who had previously been SARS-CoV-2 infected compared to those who had never been infected [30,31,32,33,34,35], suggesting that a single vaccine dose could be sufficient to enhance the natural-acquired immunity. This is most likely due to the high level of memory B cells and long-lived plasma cells, which are triggered by SARS-CoV-2 primary infection [36], as well as to “hybrid immunity” (immunity conferred by the combination of previous infection and vaccination) as suggested by Goldberg et al. [37]. 

In contrast with these results, IgG titers developed by infected-HCWs after the second dose were comparable to those observed in previously uninfected individuals, suggesting that the second dose of vaccine does not significantly boost the IgG titers in individuals with previously-acquired natural immunity. Moreover, the magnitude of the IgG response in infected-HCWs was not significantly different when the effects of the first and second vaccine dose were compared. Whereas these results are in partial contrast with previously published data [38,39,40], other studies showed that 1 month after the 2nd vaccine dose, whereas IgG antibodies binding to other spike subunits protein (e.g., S1 and S2) differed in previously infected or uninfected individuals, titers of RBD specific IgG were comparable between the two groups [41,42]. 

Interestingly, another important factor to consider is the interval between the first and the second vaccine dose, as an extended interval may increase the levels of antibodies compared to a standard 3-week interval [43,44]. In our study, it is important to note that in the case of individuals who had previously been SARS-CoV-2 infected, the first dose is actually the second “boost”. Additionally, the temporal interval between natural infection and the first dose of vaccine in our cohort is not homogeneous, with the interval being quite long (range 80–218 days) compared to the paper by Fraley et al. [41] (“recent infection” = 30–60 days). Finally, we cannot exclude that other factors (different immunoassay platforms, standardization of sample types and dilution, recent or distant previous infection) could have impacted our results: future studies are needed to clarify this aspect.

This study confirms that age affects the intensity of vaccine-induced immune responses of HCWs, with lower serum IgG concentration associated with increased age. Thus, IgG titers were inversely correlated with age after the first and the second BNT162b2 dose, as has been previously described [45,46,47]. Interestingly, this association was not observed when the effects of the third vaccine dose were analyzed. The impact of age on vaccine efficacy is a well-known issue [48,49]. More often, in the elderly, the outcome of infections [50,51] is more severe and may have serious clinical consequences as the efficacy of the immune response and the ability to properly respond to vaccines decline with age. Although these data have to be interpreted with caution because of the limited number (15/59; 25%) of over-sixties enrolled in our study, they once again underscore the importance of developing more immunogenic vaccines and/or different strategies to better protect older people. In contrast with previously reported results, no gender-associated differences were observed [8,52,53,54]; this nevertheless could be due to the low percentage of male subjects in our cohort (32%).

Neutralizing antibodies, targeting the critical domain RBD of S-protein, prevent viral binding to the host cell surface and are considered important markers of protection from reinfection, both in SARS-CoV-2 animal model [55,56] and in COVID-19 patients [57,58]. Previous papers showed that two doses of BNT162b2 induced robust NAb response [59,60]. In our study, we used a surrogate method to detect the neutralizing antibodies that can block SARS-CoV-2 RBD binding to ACE2 receptor; inhibitory activity (INH) results, expressed as a percentage of inhibition of binding, have previously shown in agreement with results obtained with conventional neutralization techniques using a live virus or pseudovirus [61,62]. The first vaccine dose could elicit NAb in most individuals, and inhibitory activity was significantly increased by the second vaccinal dose, beginning to decrease six months after this dose, in line with previous papers [63,64]. Inhibitory activity was highly correlated with IgG titers until 6 months after the second vaccine dose, indicating a parallel decline of IgG and NAb, in accordance with other authors [7,8]. Still, the lack of correlation was observed after a longer time (Mo9) or after a booster dose: so, although IgG showed a drastic decline over a long period after 2ndD, the neutralizing activity persisted for more time, suggesting different kinetics of these two parameters (IgG and NAb), consistent with findings of Malipiero et al. [65]; a possible explanation of this findings can be the increased affinity of antibodies after maturation of long-lived plasma cells, or that also other types of antibodies may participate to the persistence of neutralizing ability.

Once again, NAb response was significantly higher in those individuals who had been infected before vaccination. Importantly, INH was drastically increased by the third BNT162b2 dose, and even in the single individual who was still IgG and IgA seronegative after three doses of vaccine, a high neutralizing ability was detected by the end of the study. However, heterogeneous results can be observed in the literature concerning NAb kinetics [64,66,67,68], probably for different used assays, indicating that harmonization and standardization of SARS-CoV-2 antibody tests are needed to better evaluate the dynamics of NAb and IgG as well.

The effect of the BNT162b2 vaccine on mucosal immunity was analyzed by evaluating salivary IgA. Results showed that this vaccine does induce the generation of salivary IgA after the second dose and that this response was significantly more robust in infected-HCWs compared to naïve-HCWs, as already observed [25,26]. After reaching the peak one month after the second vaccine dose, IgA in saliva gradually diminished over time, being undetectable in a sizable proportion of HCWs 9 months after the second dose. Importantly, these antibodies were increased in most individuals ten days after the third vaccine dose, although without reaching the levels observed at 1 Mo, probably due to different intervals of time between the vaccine dose and sampling: these results need to be confirmed by other independent studies, as, at our knowledge, this is the first study that evaluates salivary IgA after the booster dose.

Finally, the IgA concentration in saliva was significantly lower than that measured in serum, as already reported [25,26], and these two parameters were not associated.

This preliminary study presents limitations, including the limited sample size, the limited duration of follow-up after booster dose, the lack of a group of individuals who were not vaccinated, and, finally, the lack of internal protein markers. Further studies on larger cohort and longer follow-up, extending to the vaccine-induced cellular immunity analyses, will need to verify the complex immune response to SARS-CoV-2 mRNA vaccines.

## 5. Conclusions

Taken together, these results confirm the BNT162b2 vaccine can elicit prompt and robust systemic and mucosal humoral immune responses that weaken over time but are quickly restored by vaccine boosters. These data further support the realization that current vaccinal protocols are the best way to curb the SARS-CoV-2 pandemic. However, as the humoral response to mRNA-based vaccines seems to be relatively short, the improvement and development of new vaccine strategies are necessary to reach a more stable IgG response in time.

## Figures and Tables

**Table 1:** Demographic characteristics of the individuals enrolled in the study.

	Naïve-HCWs	Infected-HCWs	Total
N	47	12	59
Gender (M/F)	15/32	4/8	19/40
Age, years (mean ± SD)	44.51 ± 13.74	49.50 ± 8.79	45.53 ± 12.98

Data are expressed as mean ± standard deviation (SD); HCWs: healthcare workers; M: male; F: female N: number.

## Data Availability

The data presented in this study are available on request from the corresponding author.

## Supplementary Materials

The following supporting information can be downloaded at: https://www.mdpi.com/article/10.3390/vaccines10101649/s1, Table S1: Serum IgG concentrations in healthcare workers vaccinated with BNT162b2 vaccine according to prior SARS-CoV-2 exposure status; Table S2: Serum IgA concentrations in healthcare workers vaccinated with BNT162b2 vaccine according to prior SARS-CoV-2 exposure status; Table S3: Detection of serum RBD-neutralizing antibodies (NAb) and inhibition activity (INH) in healthcare workers vaccinated with BNT162b2 vaccine, according to prior SARS-CoV-2 exposure status; Table S4: Salivary IgA concentrations in healthcare workers vaccinated with BNT162b2 vaccine, according to prior SARS-CoV-2 exposure status. Figure S1: Comparison of the antibody response to BNT162b2 vaccine according to prior SARS-CoV2 exposure status. IgG (panel a) and IgA (panel b) concentration in serum or IgA (panel c) concentration in saliva measured at different time points in Naïve- or Infected- healthcare workers (HCWs). Box plots display the median values with the interquartile range (lower and upper hinge), and whiskers indicate the minimum-to maximum range. Cut-offs are represented by dotted lines. AU: arbitrary units; U: units; Mo: month; 10daB: 10 days after the booster.
