# Supplementary material for: Systemic and Mucosal Humoral Immune Response Induced by Three Doses of the BNT162b2 SARS-CoV-2 mRNA Vaccines
Source: Vaccines (Basel). 2022 Oct 1;10(10):1649. doi: 10.3390/vaccines10101649 (PMC9610882; doi:10.3390/vaccines10101649)
Supplement: Supplementary file 1 [file vaccines-10-01649-s001.zip › vaccines-1898950-supplementary.pdf]

Supplementary Materials

Table S1. Serum IgG concentrations in healthcare workers vaccinated with BNT162b2 vaccine according to prior SARS-CoV-2 exposure status.

	**Naïve-HCWs**	**Infected-HCWs**	**Total**
**SARS-CoV-2 IgG (AU/ml)**	N (%)<br>median, (IQR)	N (%)<br>median, (IQR)	N (%)<br>median, (IQR)
Baseline	0/47	9/10 (90%)<br>108.08 (25.75-307.71)	9/57 (15.79%)<br>108.08 (25.75-307.71)
2nd dose	40/47 (85.11%)<br>62.02 (32.36 - 99.50)	12/12 (100%)<br>293.75 (205.62 - 343.19)	52/59 (88.14%)<br>80.95 (42.01 - 205.62)
Mo1	47/47 (100%)<br>319.96 (183.61 – 500.00)	12/12 (100%)<br>240.68 (191.23 - 293.27)	55/57 (96.49%)<br>278.80 (183.76 – 426.03)
Mo3	47/47 (100%)<br>89.74(53.38 - 124.34)	12/12 (100%)<br>90.31 (82.31 - 103.05)	59/59 (100%)<br>89.74 (54.52 - 120.67)
Mo6	34/45 (75.56%)<br>23.77 (16.91 - 34.91)	11/12 (91.67%)<br>40.62 (25.33 - 54.66)	45/57 (78.95%)<br>26.37 (18.21 - 39.69)
Mo9	20/40 (50%)<br>15.425 (12.67 - 20.90)	9/10 (90%)<br>27.66 (19.45 - 49.92)	29/50 (58.0%)<br>18.90 (13.07 - 25.01)
10daB	36/37 (97.22%)<br>143.11 (89.04 - 201.51)	9/9 (100%)<br>168.19 (70.87 - 305.37)	44/45 (97.78%)<br>147.14 (88.38 - 214.13)

N: number of subjects with detectable IgG in serum and prevalence in the overall sample expressed as percentage. IgG: immunoglobulin G; Mo: month; 10daB: 10 days after booster; HCWs: Healthcare workers; IQR: interquartile range; SARS-CoV-2: Severe acute respiratory syndrome Coronavirus 2; AU: arbitrary Units.

Table S2. Serum IgA concentrations in healthcare workers vaccinated with BNT162b2 vaccine according to prior SARS-CoV-2 exposure status.

	**Naïve-HCWs**	**Infected-HCWs**	**Total**
**SARS-CoV-2 IgA (U/ml) in serum**	N (%)<br>median, (IQR)	N (%)<br>median, (IQR)	N (%)<br>median, (IQR)
Baseline	0/47	9/10 (90%)<br>40857.3 (8915.3-86146.2)	9/57 (15.79%)<br>40857.3 (8915,33 - 86146,19)
2nd dose	21/47 (44.68%)<br>5923.9 (2160.2-25075.7)	12/12 (100%)<br>30338.4 (13518.0-56651.3)	23/59 (38.98)<br>15250.1 (3992,12 - 39514,57)
Mo1	42/47 (89.36%)<br>15307.5 (7555.8-35570.1)	12/12 (100%)<br>50072.3 (23708.9-96521.0)	54/59 (91.52%)<br>18436.4 (9155,06 - 55847,75)
Mo9	14/40 (35%)<br>7468.1 (1971-14250.8)	9/10 (90%)<br>45886.2 (6030.1-67595.0)	23/50 (14.18%)<br>9711.6 (2709,78 - 23139,37)
10daB	32/37 (86.48%)<br>32290.7 (14689.7-53137.0)	10/11 (90.90%)<br>122324.7 (17818.2-214937.4)	42/48 (87.50%)<br>33811.1(16356,27 - 84070,32)

N: number of subjects with detectable IgA in serum and prevalence in the overall sample expressed as percentage. IgA: immunoglobulin A; Mo: month; 10daB: 10 days after booster; HCWs: Healthcare workers; IQR: interquartile range; SARS-CoV-2: Severe acute respiratory syndrome Coronavirus 2; U: units.

Table S3. Detection of serum RBD-neutralizing antibodies (NAb) and inhibition activity (INH) in healthcare workers vaccinated with BNT162b2 vaccine, according to prior SARS-CoV-2 exposure status.

	**Naïve-HCWs**	**Infected HCWs**	**Total**
	N (%)<br>INH, median, (IQR)	N (%)<br>INH, median, (IQR)	N (%)<br>INH, median, (IQR)
Baseline	0/45	7/10 (70%)<br>93.24 (79.38 – 97.70)	7/55 (12.72%)<br>93.24 (79.38 – 97.70)
2nd dose	35/45 (77.78%)<br>56.02 (45.55-77.16)	12/12 (100%)<br>97.71 (97.40-97.82)	47/57 (82.5%)<br>68.21 (48.75-97.36)
Mo1	46/46 (100%)<br>96.71 (94.79-97.36)	12/12 (100%)<br>97.58 (97.49-97.76)	58/58 (100%)<br>96.96 (95.52-97.48)
Mo3	47/47 (100%)<br>94.89 (90.61-96.43)	12/12 (100%)<br>96.74 (95.50-97.21)	59/59 (100%)<br>95.31 (92.19-96.65)
Mo6	42/45 (93.33%)<br>84.58 (58.14-93.60)	12/12 (100%)<br>97.06 (96.57-97.74)	54/57 (94.7%)<br>87.93 (66.97-96.40)
Mo9	35/40 (87.50%)<br>65.47 (49.48-73.43)	10/10 (100%)<br>74.38 (73.07-87.84)	45/50 (90%)<br>69.44 (52.63-74.97)
10daB	37/37 (100%)<br>83.66 (75.26-96.78)	9/9 (100%)<br>96.90(89.29-96.97)	46/46 (100%)<br>95.38 (75.49-96.90)

N: number of subjects with detectable NAb in serum and prevalence in the overall sample expressed as percentage. INH: inhibitory activity in subjects with NAb in serum. Mo: month; 10daB: 10 days after booster; HCWs: Healthcare workers; IQR: interquartile range; SARS-CoV-2: Severe acute respiratory syndrome Coronavirus 2.

Table S4. Salivary IgA concentrations in healthcare workers vaccinated with BNT162b2 vaccine, according to prior SARS-CoV-2 exposure status.

	**Naïve-HCWs**	**Infected HCWs**	**Total**
**SARS-CoV-2 IgA (U/ml) in saliva**	N (%)<br>median, (IQR)	N (%)<br>median, (IQR)	N (%)<br>median, (IQR)
Baseline	0/47 (0%)	6/8 (75%)<br>999.11 (758.81 – 1321.05)	6/55 (10.91%)<br>999.11 (758.81 – 1321.05)
2nd dose	40/46 (86.96%)<br>209.37 (130.28-313.25)	11/12 (91.67%)<br>434.42 (215.44-713.63)	51/58 (87.93%)<br>219.24 (134.35-382.18)
Mo1	44/46 (96.65%)<br>365.6 (250.03-480.26)	11/11 (100%)<br>575.60 (318.27-790.32)	55/57 (96.49%)<br>384.34 (268.51-569.81)
Mo3	46/46 (100%)<br>221.93 (146.10-296.88)	11/11 (100%)<br>349.24 (272.30-502.07)	57/57 (100%)<br>235.99 (151.76-329.28)
Mo6	45/45 (100%)<br>127.35 (87.50-195.53)	12/12 (100%)<br>217.30 (112.16-338.98)	57/57 (100%)<br>138.69 (103.51-222.42)
Mo9	23/40 (57.50%)<br>61.18 (40.03-102.20)	10/10 (100%)<br>111.37 (76.91-135.81)	33/50 (66.0%)<br>76.91 (45.65-107.57)
10daB	35/37 (97.22%)<br>92.69 (53.20-136.90)	9/9 (100%)<br>162.38 (130.73-197.78)	44/46 (97.8%)<br>110.59 (53.47-160.21)

N: number of subjects with detectable IgA in saliva and prevalence in the overall sample expressed as percentage. IgA: immunoglobulin A; Mo: month; 10daB: 10 days after booster; HCWs: Healthcare workers; IQR: interquartile range; SARS-CoV-2: Severe acute respiratory syndrome Coronavirus 2; U: units.

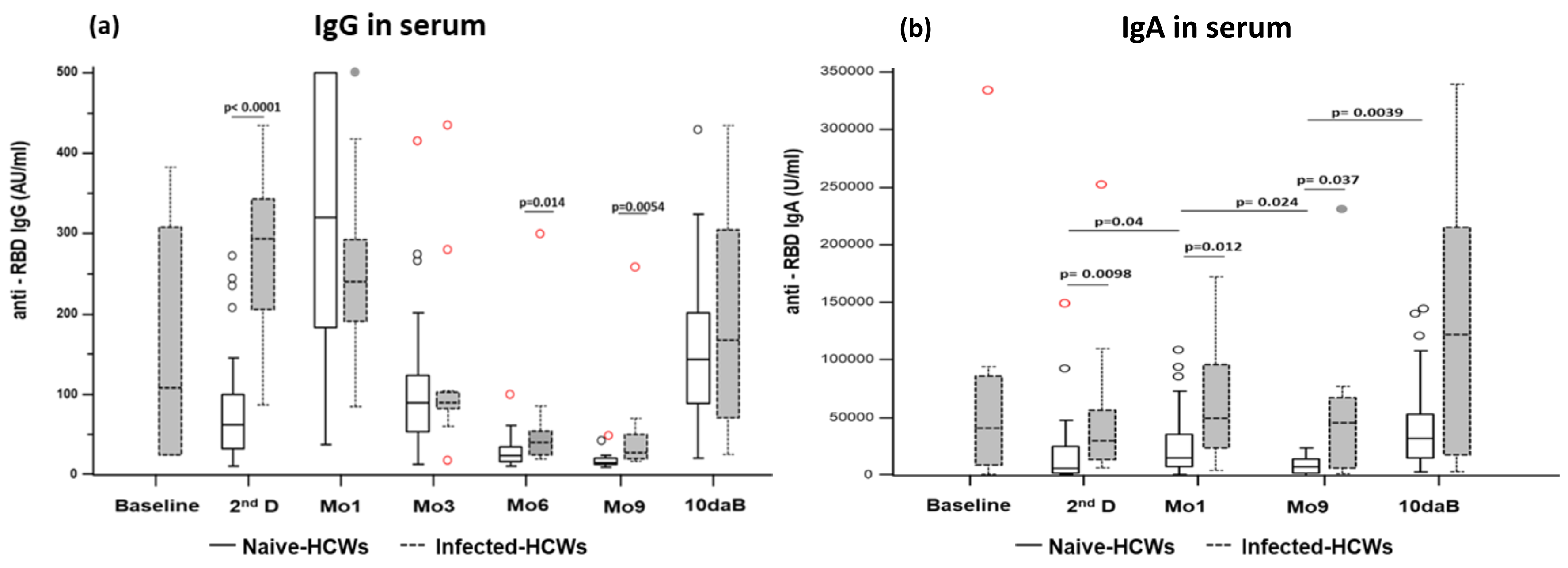

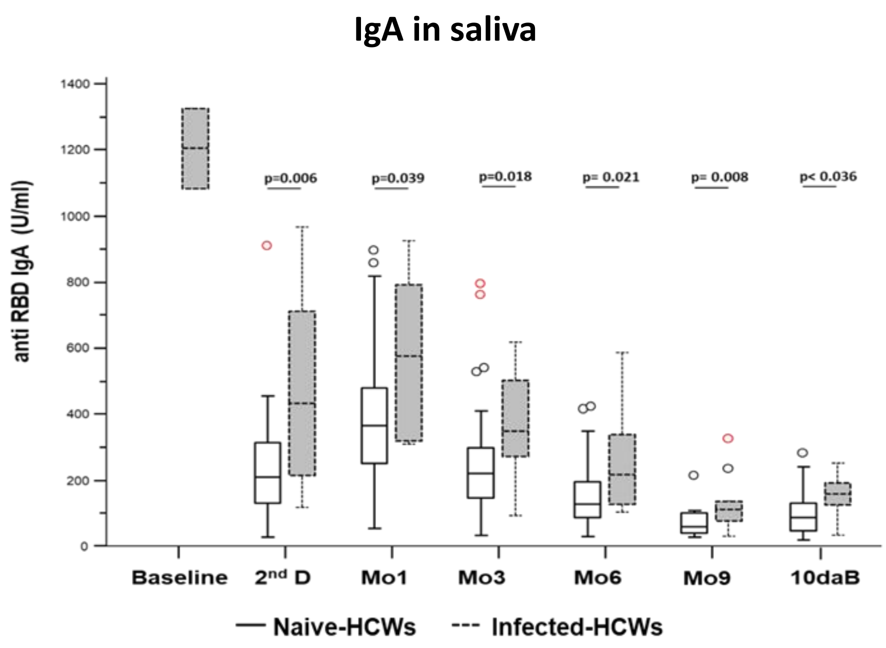

Figure S1. Comparison of the antibody response to BNT162b2 vaccine according to prior SARS-CoV2 exposure status. IgG (panel a) and IgA (panel b) concentration in serum, or IgA (panel c) concentration in saliva measured at different time-points in Naïve- or Infected- healthcare workers (HCWs). Box plots display the median values with the interquartile range (lower and upper hinge), and whiskers indicate the minimum-to maximum range. Cut-off are represented by dotted lines. AU: arbitrary units; U: units; Mo: month; 10daB: 10 days after booster.
